# A Heuristic Approach for Optical Transceiver Placement to Optimize SNR and Illuminance Uniformities of an Optical Body Area Network

**DOI:** 10.3390/s21092943

**Published:** 2021-04-22

**Authors:** Komal Masroor, Varun Jeoti, Micheal Drieberg, Sovuthy Cheab, Sujan Rajbhandari

**Affiliations:** 1 Department of Electrical and Electronic Engineering, Universiti Teknologi PETRONAS, Seri Iskandar, Perak 32610, Malaysia; mdrieberg@utp.edu.my; 2 Department of Electrical and Electronic Engineering, NED University of Engineering & Technology, Karachi 75270, Pakistan; 3 Faculty of Technical Sciences, University of Novi Sad, 21000 Novi Sad, Serbia; varunjeoti@uns.ac.rs; 4 SmartLiFi, Coventry CV6 4NQ, UK; ac1378@coventry.ac.uk

**Keywords:** illuminance, optical body area network (OBAN), optimal lamp arrangement, reciprocity, signal-to-noise ratio (SNR) uniformity, visible light communication (VLC)

## Abstract

The bi-directional information transfer in optical body area networks (OBANs) is crucial at all the three tiers of communication, i.e., intra-, inter-, and beyond-BAN communication, which correspond to tier-I, tier-II, and tier-III, respectively. However, the provision of uninterrupted uplink (UL) and downlink (DL) connections at tier II (inter-BAN) are extremely critical, since these links serve as a bridge between tier-I (intra-BAN) and tier-III (beyond-BAN) communication. Any negligence at this level could be life-threatening; therefore, enabling quality-of-service (QoS) remains a fundamental design issue at tier-II. Consequently, to provide QoS, a key parameter is to ensure link reliability and communication quality by maintaining a nearly uniform signal-to-noise ratio (SNR) within the coverage area. Several studies have reported the effects of transceiver related parameters on OBAN link performance, nevertheless the implications of changing transmitter locations on the SNR uniformity and communication quality have not been addressed. In this work, we undertake a DL scenario and analyze how the placement of light-emitting diode (LED) lamps can improve the SNR uniformity, regardless of the receiver position. Subsequently, we show that using the principle of reciprocity (POR) and with transmitter-receiver positions switched, the analysis is also applicable to UL, provided that the optical channel remains linear. Moreover, we propose a generalized optimal placement scheme along with a heuristic design formula to achieve uniform SNR and illuminance for DL using a fixed number of transmitters and compare it with an existing technique. The study reveals that the proposed placement technique reduces the fluctuations in SNR by 54% and improves the illuminance uniformity up to 102% as compared to the traditional approach. Finally, we show that, for very low luminous intensity, the SNR values remain sufficient to maintain a minimum bit error rate (BER) of $10^{-9}$ with on-off keying non-return-to-zero (OOK-NRZ) modulation format.

## 1. Introduction

**Figure 1:**
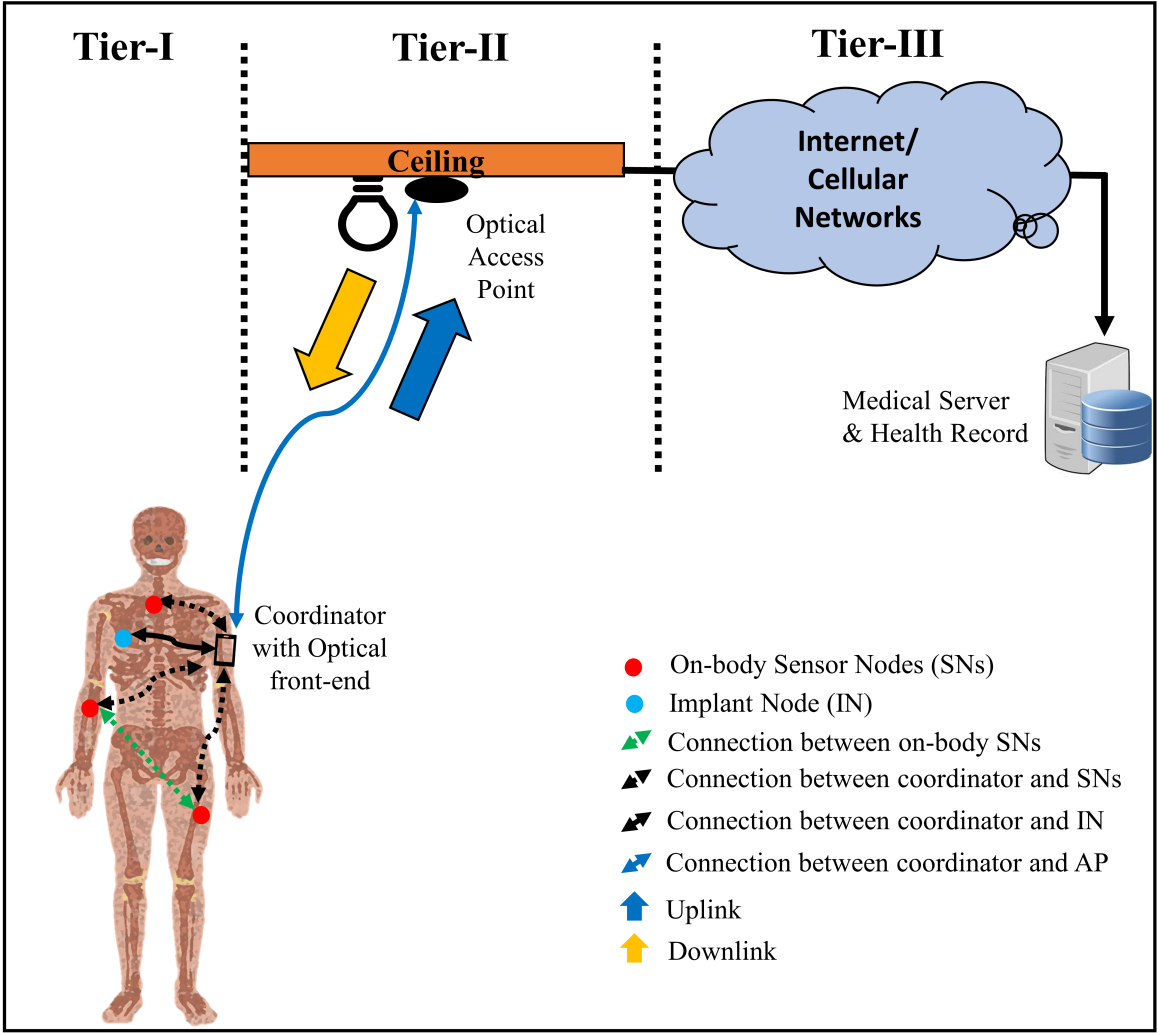
Indoor OBAN communication architecture.

Radio frequency (RF) based wireless technologies, such as Bluetooth, Zigbee, and Wi-Fi, have been in use for decades in various short- and long-range communication applications which employ frequencies in the unregulated Industrial, Scientific, and Medical (ISM) band. Recently, its widespread use can be attributed to the emergence of internet-of-things (IoT), particularly with its application in healthcare. Medical Body Area Network (MBAN) is one such example, which is a network of sensor nodes (SNs) placed in-, on-, or around a patient’s body to transmit and receive information wirelessly. The Federal Communications Commission (FCC) has already allocated a spectrum of 2360–2400 MHz band for monitoring patient’s data while using the healthcare capability of the MBAN devices [1]. However, the spectrum-hungry demands of healthcare-based IoT applications will soon find that these RF carriers are inadequate to meet the escalating needs. Currently, visible light communication (VLC) is considered to be an alternate technology to complement the overcrowded RF spectrum for many applications, including MBANs [2,3]. Accordingly, these networks are termed as Optical Body Area Networks (OBANs). With its large unregulated bandwidth, which is almost 10000 times greater than the entire RF spectrum, optical technology offers higher data rates and lower costs. Additionally, the optical links do not incur electromagnetic interference and multipath fading. Nevertheless, when these links are established in an indoor environment, they experience multipath dispersion, leading to inter-symbol interference (ISI) at high data rates. However, even the non-directed line-of-sight (LoS) optical links support data rates up to few hundred Mbps (esp. 10 kbps–10 Mbps that are required for patient monitoring [4]); hence, multipath effects are not significant in OBANs [3,5]. As illustrated in Figure 1, an OBAN generally uses the three-tier communication architecture of MBANs [4], which comprises of tier-I, tier-II, and tier-III communications as described below:

*Tier-I or intra-BAN Communication:* is the type of communication that occurs between in- or on-body SNs and the sink (also called hub or coordinator). The former collects physiological parameters from the body and transmits them to the latter via a wired or wireless medium. While it is also possible for the SNs to communicate with each other, ultimately it is the coordinator that receives information from the SNs, processes it, and then sends it to the next level, i.e., tier-II.

*Tier-II or inter-BAN Communication:* is responsible for establishing a bi-directional connection between the hub and an external/ off-body device, e.g., an access point (AP), so that the data collected at tier-I can be forwarded to the remote station at tier-III (see Figure 1). Here, UL communication occurs when the on-body coordinator sends data collected from tier-I to an AP located in the patient’s vicinity, whereas DL communication happens when the coordinator receives information from the AP. Generally, in OBANs, infrared (IR) is used for UL whereas visible light (VL) is used for DL communication.

*Tier-III or Beyond-BAN Communication:* uses Internet or cellular (peer) networks to transfer patient-related information from AP/ gateway to the medical server (MS), as illustrated in Figure 1. Moreover, this component is responsible for processing, analyzing, and storing the information in a database server to maintain an electronic medical history of the patients as well as for decision making, e.g., to trigger alarms or prompt the emergency services in case any anomaly is observed in the data; for instance, when a parameter under observation increases above a threshold level.

Although the communication hierarchy in OBANs is similar to that of MBANs, yet, instead of the conventional RF-based devices, it employs optical transmitters (Tx) and receivers (Rx) to create a communication network. Hence, the connections between nodes are optical in nature and they rely on non-coherent transmission and detection techniques, i.e., Intensity Modulation and Direct Detection (IM/DD). Additionally, depending on the Tx directivity and Rx field-of-view (FOV), these optical connections can broadly be classified into LoS and non-LoS (NLoS). While directed LoS links depend on accurate pointing between transceivers, they do so at the cost of mobility, whereas the NLoS (diffuse) links support mobility and do not require strict alignment [5]. Using these broad link configurations, two types of connections can be established in OBANs for UL and DL communication, i.e.: (i) between coordinator and AP (tier- II) and (ii) amongst on-body sensors (tier-I). These two cases correspond to MBAN channels identified as S4 to S7 according to IEEE ratified 802.15.6 standard [4]. Even though the two-way data transmission and reception in OBANs are critical at all the three tiers of communication, UL and DL connections at tier-II are of utmost importance because they not only carry patients’ sensitive information, but also establish a link between tier- I and tier- III. Furthermore, the QoS of the bi-directional UL and DL wireless transmissions encompasses robustness and reliability with the bit error rate (BER) expected to be $10^{-9}$ or less [4,6,7], for instance, in applications like ECG, EEG, drug delivery, and glucose level monitoring [7]. Thus, any negligence at this level could be life-threatening and, so, QoS remains a fundamental design issue at tier-II. Accordingly, it is imperative that the data collected by the coordinator from the SNs at tier- II must be transmitted/ received reliably to/from the AP and vice versa to avoid any mishaps. While reliability in OBANs depends on several factors, such as blocking effects [6], ambient light interference [8], and mobility [9], broadly, it primarily relies on the received SNR levels at the photodetector (PD) which in turn determine the system performance. For a typical OBAN employing IM/DD with OOK-NRZ modulation format, the required theoretical SNR is 15.6 dB to maintain a minimum (threshold) BER of $10^{-9}$[6,10]. This implies that a minimum SNR of 15.6 dB must be made available throughout the room to ensure desired communication performance for OBANs. Hence, the ultimate challenge for an indoor OBAN designer is not only to maximize the value of SNR at the receiver, but also to maintain nearly uniform SNR at all points in the room, regardless of the Tx or Rx positions for UL and DL, respectively.

The SNR is principally determined by the channel’s direct current (DC) gain. While, in an OBAN link, the channel characteristics are fixed for a given position of transmitters, receivers, and intervening reflecting objects, they tend to change when these components are moved by distances in the order of centimeters [10]. Therefore, changing the transmitter positions will significantly impact the overall communication performance. Additionally, transmitter placement also plays a crucial role in obtaining the uniform distribution of illuminance, which determines the received power ($P_{r}$) and, subsequently, the SNR. On the contrary, the non-uniform distribution may not only be displeasing aesthetically, but it also leads to a high degree of fluctuations in the received SNR. These fluctuations restrict the system’s ability to provide equal communication quality to users, especially in the corners of the room. Hence, it leads to poor communication quality and an unreliable link. Currently, the widely adopted placement technique [2,11] in the literature fails to provide seamless user experience when it comes to reducing the SNR fluctuations. As the ultimate aim in an OBAN is to establish reliable bi-directional communication, it is critical to analyze and optimize the transmitter locations to improve SNR and illuminance uniformities of the DL connection. Moreover, since the transmission of patient’s data via UL is more crucial than the DL, therefore, SNR uniformity must also be retained for UL, irrespective of the patient’s posture and his/ her position inside the room.

In our previous work [12], we analyzed the effects of transmitter layout on illuminance uniformity for which we studied three different scenarios, i.e.: (a) single transmitter, (b) the generalized centrally lit quadrants (CLQ) with four transmitters, and (c) the proposed scheme of relocating all four transmitters towards each corner of the room referred to as corner illuminated quadrants (CoIQ). The single transmitter scenario offers a significant variation in SNR and reliable communication is not feasible. The proposed cornered LED lamp placements provided better results than the conventional four transmitter quadrant lit scenarios. Moreover, in both cases, there remained an area with low illuminance, known as the blind spot, which significantly degraded the illuminance uniformity. To eradicate this issue, a fifth transmitter was added to the system in [13]. Currently, there are several different lamp placement designs available in the literature, for instance, with five [13], six [14], and sixteen [15] luminaires. Even though the addition of transmitters may improve SNR and illuminance uniformities, it comes at the expense of cost, increased root mean square (RMS) delay spread, and reduced bit rates [16]. Accordingly, this work aims to increase the DL SNR and illuminance uniformities by heuristically optimizing the transmitter locations with the minimum number of transmitters. Subsequently, we use these optimum positions to achieve reduced SNR fluctuations; hence, a reliable link with a minimum BER of $10^{-9}$ at a data rate of 20 Mbps for OOK-NRZ modulation format. Besides, the application and validation of the principle of reciprocity (POR) is presented. It is assumed that, while using POR [17], this DL analysis will also ensure the UL communication quality in terms of improved SNR uniformity throughout the indoor coverage area. To the best of the authors’ knowledge, the characterization of a UL connection based on optimized DL transmitter locations has not been reported in the literature. The main contributions of this study are listed below:

- OBAN DL connection is characterized by analyzing the impact of transmitter locations to improve SNR and illuminance uniformities and to enable QoS support.
- A simple and heuristic placement scheme is proposed for generalized square room geometry that guarantees better performance when compared to the traditional approach.
- An algorithm is developed that, given the room dimensions, yields optimized coordinates with minimum fluctuations in SNR over a resolution of 0.25 m.
- A comparative study on placement schemes and validation of the proposed heuristic design formulae is also presented by analyzing twelve different placement scenarios.
- Using the principle of reciprocity and based on the proposed DL transmitter and receiver locations, UL SNR uniformity is estimated and validated.

The rest of the paper is organized, as follows: Section 2 covers the related work followed by a comprehensive system description in Section 3. A presentation of results and their elaboration is available in Section 4. Finally, Section 5 concludes this paper.

## 2. Related Work

A VLC system mostly utilizes the illumination infrastructure for communication. Even though minor fluctuations in illumination within a room are acceptable for general lighting, uniform illumination must be attained to provide full room coverage, avoid significant variations in SNR, and guarantee a reliable link. Hence, uniform lighting conditions, which refer to evenly distributed lighting across a room, are critical from the communication perspective, since the $P_{r}$ profile at the PD corresponds to the values of illuminance [14]. Additionally, illuminance uniformity is also essential to ensure smooth coverage over any given area. Therefore, the optimum LED lamp layout can play a crucial role in improving the system performance.

The transmitter locations can either be evaluated by using illuminance or $P_{r}$ [18]. There are a few studies available in the literature which have examined the effects of lamp locations and its number on the uniformity of illuminance, for instance, in [19], an optimized layout was obtained to maximize the average area spectral efficiency for more than one light fixtures using the Genetic algorithm. In [20], the effects of different layouts were analyzed by considering simplified and non-simplified sources. In [15], a layout was presented to minimize the fluctuations in SNR in the presence of multiple users. In [21], the authors presented an inverse design to determine the optimal arrangement while using a convex optimization algorithm. Nevertheless, most of these studies either used more than five transmitters or utilized complex optimization algorithms to obtain optimum transmitter locations. Besides, these studies only accentuated the impact of LED lamp layouts on the uniformity of illuminance and $P_{r}$ of a generalized VLC set-up, e.g., high-speed internet. However, an OBAN system has stringent QoS requirements in terms of reliability, where any negligence could result in a lethal event. Hence, OBANs are different from the regular optical-based wireless network infrastructure and must be investigated separately. For instance, when using VLC for patient monitoring, the subject wearing the on-body receiver could have various postures that tend to alter the receiver height. Because $P_{r}$ is inversely proportional to the square of the distance between the transmitters and receivers, varying heights may affect it accordingly. Additionally, the body also introduces some loss, depending on the on-body receiver location. Consequently, these additional losses must also be taken into account when dealing with patient monitoring.

The performance of VLC and/or IR links in OBANs has been under investigation for over a decade now; nevertheless, its feasibility study was first presented by Torkestani et al in [22]. The authors used diffused IR technology to monitor patients’ vital signs and developed a model of the mobile channel in 2D and 3D scenarios for on-off keying (OOK) modulation. Moreover, they demonstrated that optical technology could potentially be used as an alternative to RF in patient monitoring systems. Later, several studies exploited the use of VLC technology for healthcare data transmission. For instance, diffuse VLC links in healthcare scenarios in the presence of ambient light interference were experimentally demonstrated in [8] and they achieved a BER of $10^{-5}$ for 10 Mbps transmission. In [23], white-light LEDs were used to successfully transmit biomedical data and healthcare information. In [6], the blocking effects were considered to showcase their impact on OBAN communication. While all of these studies focused on a single-user environment, Refs. [9,24,25] discussed the use of VLC in multi-user environments. Recently, the authors in [26] characterized the intra-OBAN channels by considering the effects of mobility and shadowing that occurred as a result of body parts and realistic body movements. In another study [27], the authors have reported the effects of several parameters, e.g., source directivity, transmitted optical power, position of on-body sensors, and the number of PDs that were installed on the ceiling. However, in all of these OBAN related studies, mainly the UL connection has been investigated and the impacts of fixed transmitter and receiver locations on the DL and UL communication, respectively, have been ignored. Accordingly, this study emphasizes investigating the effects of LED arrangements while taking into account the body losses (BL) as well as the patient’s posture to estimate and ensure QoS availability for DL and UL connections.

## 3. System Description

This study has the following main objectives:

1. Develop a heuristic design formula to achieve optimum transmitter locations for a square room geometry; hence, optimize the illuminance and SNR uniformities with a minimum number of transmitters
2. Achieve a BER of $10^{-9}$ (or less) for the OOK-NRZ modulation scheme to ensure an unbroken and reliable communication link with the received SNR > 15.6 dB in worst-case low-intensity scenarios

By determining the optimum positions of the LED transmitters for given room dimensions, it would be ensured that the illuminance uniformity is optimized, $P_{r}$ is evenly distributed, the fluctuations in SNR are minimum, and the least SNR value is well above the defined SNR threshold. To realize these goals and present our results in the next section, this segment covers the description of generalized methodology, system and channel models, proposed LED design layout algorithm, and other related material required to carry out the ray-tracing simulations.

### 3.1. Methodology

**Figure 2:**
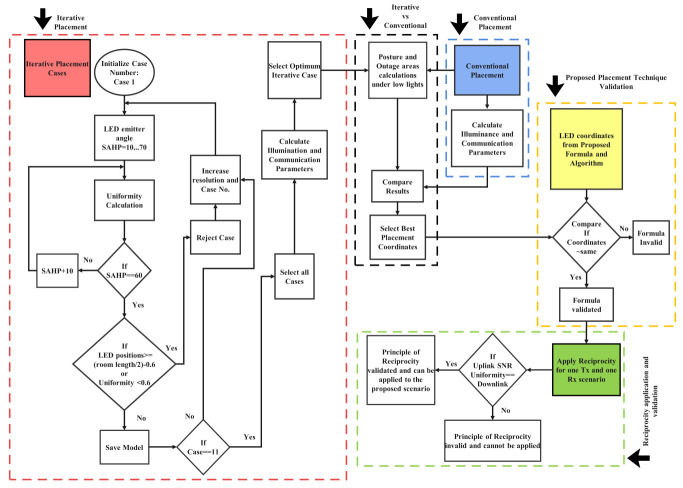
An overview of the Methodology adopted in this work to achieve signal-to-noise ratio (SNR) and illuminance uniformities.

An overview of the methodology adopted in this study is illustrated in Figure 2. It comprised of five main parts:

#### 3.1.1. Iterative Placement Analysis

**Figure 3:**
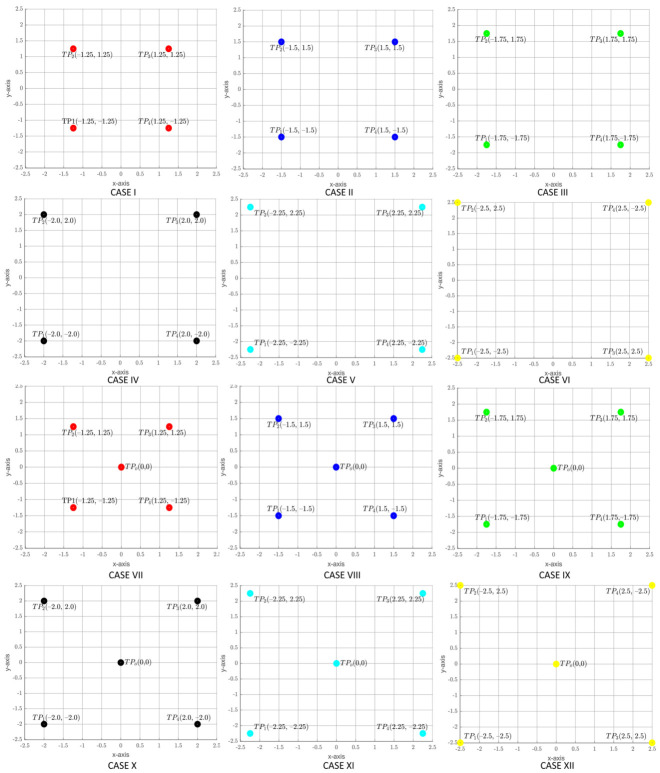
LED lamps placement scenarios: Cases I to XII.

This part considered eleven different iterative lamp arrangements to determine the optimum transmitter locations based on uniformity and general lighting conditions. Even though the iterations could yield several placement combinations, the eleven candidate scenarios were obtained by starting at the conventional placement locations that are defined by Cases I and VII in Figure 3 for four and five transmitters, respectively, and then moving symmetrically along the room diagonals with a resolution of 0.25 m to generate further cases until the ends of the diagonals were reached. The placements that fit the uniformity and lighting criteria were then used to calculate illuminance and communication parameters. Subsequently, the case with minimum SNR fluctuations was compared with the conventional technique.

#### 3.1.2. Conventional Placement

Being represented by Case VII in Figure 3, it has been used as the traditional placement approach (CLQ) throughout this study to establish a comparison with the proposed technique. Additionally, this is the part where the illuminance and communication parameters of CLQ were calculated. It was run in parallel to the iterative analysis, so, altogether, there were twelve cases under consideration (see: Figure 3).

#### 3.1.3. Iterative vs. Conventional Placement

At this stage, CLQ and the best case with minimum SNR fluctuations that were obtained from Section 3.1.1 were used to perform posture analysis under low-lighting conditions and to obtain the percentage of outage areas for each case. The optimal arrangement that was retrieved with better BER support and availability was then compared with placement obtained in Section 3.1.4.

#### 3.1.4. Proposed Formulae and Algorithm Validation

Instead of using the iterative approach (as described in Section 3.1.1), this stage involved the generation of optimized transmitter locations via the proposed Algorithm 1. Next, its validation was performed by comparing these positions to the best case acquired from Section 3.1.3. If the values were approximately same, the proposed technique was considered to be valid, and vice versa.
**Algorithm 1** Algorithm to determine coordinates’ for given room size using the proposed formulae.**Input** $\mapsto \;L_{x}\;,L_{y}\;$ and $\;L_{z}$**1. Initialize**$\;\;Illuminance\leftarrow {\left[0\right]}_{N_{x}\times N_{y}}$$\;\;ReceivedPower\left(P_{r}\right)\leftarrow {\left[0\right]}_{N_{x}\times N_{y}}$$\;\;SNR\leftarrow {\left[0\right]}_{N_{x}\times N_{y}}$Calculatediagonalpoints$\;\;Define\;positions:\;TP_{0},\;TP_{1},\;TP_{2},\;TP_{3},\;and\;TP_{4}$**2. Calculate** ↦ Illuminance uniformity for all TPs**for all** Rows($N_{x}+1$) of $P_{r}$
**do** **for all** Columns($N_{y}+1$) of $P_{r}$
**do**  
$\;P_{r}\left(r1,c1\right)\leftarrow P_{r}\left(N_{x}+1,N_{y}+1\right)$  
$\;r1\leftarrow N_{x}+2$  
$\;c1\leftarrow N_{y}+2$ **end for****end for** 
$SNR\leftarrow Calculate\;SNR\;for\;all\;values\;of\;P_{r}$CalculatestandarddeviationofSNR**3. Repeat** ↦ Step 2 for $(TP_{0},\;TP_{1},\;TP_{2},\;TP_{3},\;and\;TP_{4})\;+▵r$**4. Repeat** ↦ Step 2 for $(TP_{0},\;TP_{1},\;TP_{2},\;TP_{3},\;and\;TP_{4})\;-▵r$**if** 
$min(\sigma_{SNR}\in TP_{n}\left(x_{n},y_{n}\right))$ 
**then** 
$display\;TP_{n}\left(x_{n},y_{n}\right)$**else if** 
$min(\sigma_{SNR}\in TP_{n}\left(x_{n},y_{n}\right))+▵r$ 
**then** 
$display\;TP_{n}\left(x_{n},y_{n}\right)+▵r$**else if** 
$min(\sigma_{SNR}\in TP_{n}\left(x_{n},y_{n}\right))-▵r)$ 
**then** 
$display\;TP_{n}\left(x_{n},y_{n}\right)-▵r$**end if**

#### 3.1.5. Application and Validation of the Principle of Reciprocity

Because it has been proposed in this study that the UL SNR uniformity can be assessed from the DL results by using POR, the last part focused on demonstrating this principle when applied to a single Tx and single Rx scenario. If the UL SNR uniformity matched the DL value, then the principle was considered to be valid and it could be applied to estimate the UL performance as proposed.

### 3.2. System Model

This section covers the channel, room, reflector, and human body models that were used in this study to simulate the indoor OBAN system environment.

#### 3.2.1. Room and Reflector Models

**Figure 4:**
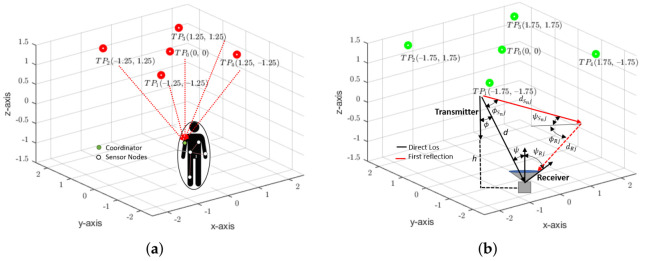
LED lamp placements for (**a**) centrally lit quadrants (CLQ) and (**b**) corner illuminated quadrants (CoIQ).

Figure 4a illustrates the OBAN system model that was considered in this study. The tier-II DL connection comprises of five VLC transmitters (TP${}_{1}$, TP${}_{2}$,…TP${}_{5}$) distributed on the ceiling, where TP${}_{n}$ (x,y,z) denotes the transmitter position with coordinates (x,y,z) in meters and ‘*n*’ represents the transmitter number. Based on [13], an LED array of 50×50 is used for each luminaire. The DL receiver is assumed to be a 1 cm${}^{2}$ silicon-based positive-intrinsic-negative (PIN) PD placed on the patient’s arm. Conversely, for the UL connection, an on-body IR transmitter is placed together with the DL VLC detector, whereas the IR receivers are assumed to be placed on the ceiling alongside the DL transmitters represented by (RP${}_{1}$, RP${}_{2}$, …RP${}_{5}$). To establish an OBAN link between the Txs and Rx, the most performing link configuration is the LoS; nevertheless, a perfect LoS connection may not always be feasible due to random human body movements. Alternately, since, non-directed LoS links can support mobility scenarios in indoor environments; therefore, this study employs LoS with non-directed first-order reflections (see Figure 4b). Accordingly, it is assumed that the patient could be present at any random location along the XY-plane in the indoor space (see Figure 4). Besides, even though the receiver height may vary with the posture under consideration, it is assumed to be at the desktop level of 0.85 m, unless stated otherwise.

To perform the ray-tracing simulations, a room that has often been used in the literature with the dimensions of 5×5×3 m${}^{3}$ has been considered [9,11]. Given the typical low data-rates that are used in OBANs, the room furniture has a negligible impact on the main characteristics of the channel [28,29]; hence, it is not considered in the study. However, all of the lateral room surfaces are modeled as the first order Lambertian reflectors, which bear a constant reflection coefficient. Besides, to study the effects of LED lamp arrangements, two placement techniques have been considered, namely, CLQ envisaged for attocells in [13] (Figure 4a) and the proposed-CoIQ (Figure 4b). CLQ employs four transmitters that are placed at the center of each quadrant in the room with an additional LED lamp located at the geometrical center of the room itself. Conversely, CoIQ considers placing the LED lamps around each corner of the room with one transmitter that is located in the center.

#### 3.2.2. Human Body Model

A study that is based on the impact of the human body on VLC has already been presented in [30]. It has been shown that applications requiring high-QoS must consider the effects of human body reflectivity and data rates. Furthermore, it explains that a body possessing high reflectivity value (e.g., 0.7) does not significantly affect the SNR for data rates lower than 250 Mbps. Even though the human body reflectivity is highly subjected to clothing that is worn by the people and low reflectivity values must also be considered, yet, the inference for low reflectivity values, e.g., 0.1, remains the same, except that the results are more penalizing in terms of increased loss. As the aim of this study is to establish illuminance and SNR uniformities at various receiver heights for a data rate of 20 Mbps which is much less than 250 Mbps, the following assumptions are made in the DL analysis: (i) the on-body coordinator (receiver) is placed around the patient’s arm, such that it is facing the ceiling at all times except when considering the sleeping posture where it is facing the wall, and (ii) the human body has a high reflectivity value. The effects of low reflectivity values will be evaluated in a separate study. Besides, another major issue that is associated with human body modelling is that, when the receiver is worn by a moving person, it may incur random orientations. Moreover, it is likely that the receiver may only collect reflected optical paths; thereby, resulting in ISI. These issues may adversely impact the communication performance by inducing optical path losses and significantly reducing the achievable data rates. While generally the receivers are assumed to be oriented towards the ceiling, a study presented in [31] has considered the impacts of receiver orientations and positions on VLC link performance revealing that even though the effects of receiver orientations in a mobile VLC link are significant, for data rates lower than 30Mbps, they do not have a strong impact. This is because additional excess delay resulting from one or more reflections does not add to ISI problem and data rates up to 30 Mbps are supported. Since the healthcare applications generally require data rates lower than 1 Mbps [32] and, because our study is limited to a maximum of 20 Mbps, the effects of receiver orientations will not be considered.

#### 3.2.3. Channel Model

As explained earlier, OBANs employ IM/DD for transmission and reception of data, respectively. For IM/DD, the received optical signal is given by:

(1) y(t)=H(0)γx(t)+n(t)

where y(t) is the received signal, H(0) is the static channel gain, γ is the receiver responsivity, x(t) is the transmitted signal, and n(t) denotes the additive white gaussian noise (AWGN) with double-sided noise power spectral density (PSD), $N_{0}$. The DC channel gain, H(0), of an optical link is a key factor in determining $P_{r}$ which can be calculated for LoS and NLoS links using (2) and (3), respectively:

(2) $$H_{los}\left(0\right)=\frac{A_{r}\left(m+1\right)}{2\pi d^{2}}\cos^{m}\left(\phi \right)T_{s}\left(\psi \right)g\left(\psi \right)\cos\left(\psi \right)$$

(3) $${H\left(0\right)}_{nlos}^{1}=\frac{\left(m+1\right)}{2\pi }\sum_{j=1}^{\kappa }{\;\rho }_{j}\cos^{m}\left(\phi_{S_{n}j}\right)\frac{\cos\left(\psi_{S_{n}j}\right)\;cos\left(\psi_{Rj}\right)▵{\mathrm{AA}}_{r}}{d_{S_{n}j}^{2}d_{R_{j}}^{2}}$$

where $A_{r}$ is the physical area of the detector, *m* is the Lambert’s order, $T_{s}\left(\psi \right)$ is the gain of the optical filter, g(ψ) represents the concentrator gain, ▵A is the area of the differential element, κ is the total number of reflecting elements in the room, $\rho_{j}$ is the reflectivity of each differential surface, $d_{S_{nj}}$ is the distance from *n*^th^ source $S_{n}$ to reflector element j, and $d_{Rj}$ is the distance between reflector j and receiver R. The total received power ($P_{r-total}$) can then be computed as:

(4) $$P_{r-total}=\sum_{n=1}^{N_{LED}}\left(H_{los}\left(0\right)\;+\sum_{nlos}{H\left(0\right)}_{nlos}^{n}\right)P_{t}$$

The received signal quality in a VLC system can be evaluated using SNR. It is a quantity used to compare the levels of the desired signal to the level of background noise and is measured in decibels. Without incorporating the BL, the SNR is expressed as [5,33]:

(5) $${SNR}_{woBL}=\frac{{I_{sig}}^{2}\left(t\right)}{R_{b}N_{o}}=\frac{\gamma^{2}{{{H\left(0\right)}^{2}P}_{t}}^{2}}{\left({\sigma_{th}}^{2}+{\sigma_{shot}}^{2}+{\sigma_{d}}^{2}+{\sigma_{amp}}^{2}\right)B}$$

where $I_{sig}$ is the received photoelectric current, $R_{b}=B$, *B* is the bandwidth and $N_{o}$ is the summation of $\sigma_{th},\sigma_{shot},\sigma_{d}$ and $\sigma_{amp}$, which are the thermal, shot, dark, and amplifier noise PSDs, calculated using (6), (7), (8), and (9), respectively:

(6) $$\sigma_{th}^{2}=\frac{8\pi kT\xi A_{r}I_{2}B^{2}}{G}+\frac{16\pi^{2}kT\Gamma A_{r}^{2}\xi^{2}B^{3}I_{3}}{g_{m}}$$

(7) $${\sigma_{shot}}^{2}=2qI_{sig}$$

(8) $${\sigma_{d}}^{2}=2qI_{d}\;\;\;\;\;\;$$

(9) $${\sigma_{amp}}^{2}=2qI_{amp}$$

where *k* is the Boltzmann constant, *T* is the temperature in Kelvin, $I_{2}$ is the noise bandwidth factor for rectangular pulse shape, $I_{3}$ is the noise bandwidth factor for a full raised cosine and equalized pulse shape, ξ is the fixed capacitance per unit junction area, *G* is the open-loop gain, Γ is the FET channel noise factor, $g_{m}$ is the FET transconductance, *q* is the electronic charge in Coulombs, $I_{d}$ is the dark current, and $I_{amp}$ is the input-referred RMS noise. All of these parameters have been summarized in Table 1 and are derived from [5,11]. The SNR obtained from (5) is for standard VLC communication without considering the BL. Nevertheless, for patient monitoring, the SNR received at the on-body PD must also incorporate the impact of body on the communication link. Therefore, we calculate ${SNR}_{OBAN}$, as:

(10) $${SNR}_{OBAN}={SNR}_{woBL}-BL$$

Although human body does not have significant effects on the communication performance when high body reflectivity and data rates lower than 250 Mbps are considered [30], an average BL value of 5 dB is assumed in this study to account for the reflective losses that occur due to body absorption and other NLoS reflections that fall below the receiver height.

As explained earlier, the effects of transmitter locations on a system’s performance can either be determined by the study of $P_{r}$ or illuminance. The former is a radiometric quantity and can be obtained from the power transmitted by LEDs, whereas the latter is a photometric quantity that can be derived from the luminous intensity of an LED. All of the parameters described so far are radiometric, which are valid for VLC and IR; however, since VLC performs the dual purposes of communication as well as illumination, some photometric quantities must also be considered as defined in the next subsection.

#### 3.2.4. Photometric and Radiometric Quantities

Luminous intensity is an intrinsic property of LEDs which is governed by the irradiance angle (ψ). It falls off as the cosine of this angle such that [5]:

(11) $$I\left(\phi \right)=I_{o}cos\left(\phi \right)$$

where $I_{0}$ is the center luminous intensity of the LED.

Illuminance is derived from the luminous intensity. It is defined as the photometric flux incident on a surface per unit area and is measured in lux. Illuminance can have vertical as well as horizontal components. However, it is mainly characterized by the latter. Mathematically, for any point (x,y,z) in 3D space, horizontal illuminance for a single source in an array of transmitters $\left\{S=S_{1},S_{2}\ldots S_{n}\right\}$ can be expressed as [5]:

(12) $$E_{r}^{n}\left(x,y,z\right)=\left(I_{o}\cos^{m}\phi \cos\psi \right)/d_{n}^{2}$$

where ψ is the angle with which the ray is incident on the receiver, *d* represents the distance from the light source to the PD, and *m* is Lambert’s order, which defines the directivity of the source, given by:

(13) $$m=\frac{ln2}{ln\cos\phi_{\frac{1}{2}}}$$

Here, $\phi_{\frac{1}{2}}$ is the semi-angle at half power. Note that, for a transmitter and receiver lying perpendicular to the ceiling, ϕ=ψ[34] and cosϕ=h/d. For multiple transmitters, the total horizontal illuminance, $E_{T}$, is given by:

(14) $$E_{T}\left(x,y,z\right)=\sum_{n=1}^{N_{LED}}E_{r}^{n}$$

where $N_{LED}$ denotes the total number of LEDs. Interestingly, most of the studies reported in the literature, including the pioneering work by T.Komine et al. [11,13,16], have considered luminous intensity $I_{0}$ (0.73 cd/LED) and optical power $P_{t}$ (20mW/LED) as independent quantities to perform photometric and radiometric computations for illuminance and received power, respectively. However, in this study, instead of assuming the arbitrary value of 20 mW, a simplified relationship between $P_{t}$ and $I_{0}$ has been employed to establish a correspondence between these quantities for more realistic calculations. $P_{t}$ is related to $I_{0}$, as follows:

(15) $$P_{t}=I_{0}\Omega /\eta$$

where η is the luminous efficacy of LEDs in lumens per watt and Ω is the solid angle in steradian, given by:

(16) Ω=2π(1−cos(θ/2))

Here, θ is the apex angle of the cone and it is given by θ=2ϕ. The power obtained from this relationship against 0.73 cd is approximately 63 mW per LED.

### 3.3. Proposed LED Design Layout

Most of the VLC based studies in the literature typically employ a room size of 5×5×3 m${}^{3}$, wherein CLQ is generally employed for the placement of four transmitters [11]. Other studies utilize complex optimization routines to determine optimal locations for six or more transmitters. However, practically the room dimensions may vary, where CLQ based arrangements may not always provide optimum results and optimization-based algorithms are computationally intensive. Therefore, in this study, we propose the CoIQ technique and heuristically derive a simple design formula to obtain optimum transmitter locations that offer link availability at all points in the room with minimum BER of $10^{-9}$.

To determine the coordinates for a generalized VLC set-up with five transmitters, let $L_{x}$, $L_{y}$, and $L_{z}$ specify the length, width, and height of the room along the x-,y-, and z- axes, respectively. The entire 3D space is then assumed to be divided into four quadrants, such that the origin lies at the geometrical centre of the room (see Figure 4). This creates x-,y-, and z-axes that range between [−$L_{x}$/2, $L_{x}$/2], [−$L_{y}$/2, $L_{y}$/2], and, [−$L_{z}$/2, $L_{z}$/2], respectively. The XY-plane of each quadrant is then further divided into several segments with a resolution (▵r) of 0.25 m, for instance, if the length of the room is 5m then a 0.25 m resolution implies that the x- and y- axes have been virtually divided into 20 equal parts. Now, let there be five transmitters to cover the entire room where each transmitter is denoted by its transmitter position $TP_{n}\left(x_{n},y_{n},z_{n}\right)$ and ‘*n*’ refers to the number of each transmitter. $TP_{o}$ is placed at the origin with coordinates (0,0, $L_{z}$/2). However, the remaining transmitters can be defined by the diagonal points $DP_{x}$ and $DP_{y}$ along the x- and y- directions, respectively, which are calculated as:

(17) $$DP_{x}=\left(\left(L_{x}\right)*\sqrt{2}\right)/4$$

(18) $$DP_{y}=\left(\left(L_{y}\right)*\sqrt{2}\right)/4$$

Subsequently, exploiting the symmetry of the square room, the transmitters can be placed along the diagonals passing through origin with a tolerance of ±▵r, such that:

(19) $$\begin{array}{cc} TP_{1} & =[-DP_{x}\pm ▵r,\;\;-DP_{y}\pm ▵r,\;\;\;L_{z}/2]\\ TP_{2} & =[-DP_{x}\pm ▵r,\;\;\;\;DP_{y}\pm ▵r,\;\;\;L_{z}/2]\\ TP_{3} & =[\;\;DP_{x}\pm ▵r,\;\;\;\;DP_{y}\pm ▵r,\;\;\;L_{z}/2]\\ TP_{4} & =[\;\;DP_{x}\pm ▵r,\;\;-DP_{y}\pm ▵r,\;\;\;L_{z}/2] \end{array}$$

Further steps for heuristically obtaining the optimized coordinates have been summarized in Algorithm 1, which takes room dimensions $L_{x}$, $L_{y}$ and $L_{z}$ as the input. Instead of performing exhaustive iterations to acquire optimized coordinates, the algorithm then uses (17) and (18) to calculate one diagonal point ($TP(DP_{x},DP_{y}$). Next, it exploits the room symmetry to define ($TP_{0},\;TP_{1},\;TP_{2},\;TP_{3},\;$ and $\;TP_{4}$) as given in (19) and calculates the illuminance and SNR uniformities. Because (17) and (18) were devised heuristically to calculate the coordinates, it was observed that for room lengths lower than 5m, optimum results were obtained at ($DP_{x}$ + Δr, $DP_{y}$ + Δr), while for lengths greater than 5m, ($DP_{x}-\Delta r,DP_{y}-\Delta r$) produced better uniformity results. Based on these observations, steps 3 and 4 were repeated for $TP_{n}\left(x_{n},y_{n}\right)$ + Δr and $TP_{n}\left(x_{n},y_{n}\right)-\Delta r$, respectively. Accordingly, the conditional statements were used next to output the optimized coordinates based on minimum SNR deviation.

### 3.4. Statistical Parameters for Assesment of Illuminance and SNR Uniformities

The evaluation of uniformity of illuminance is mainly based on two unitless statistical quantities, namely, uniformity ($U_{o}$) and interquartile range (IQR). $U_{o}$ is the ratio of minimum ($E_{min}$) to the averaged illuminance value $\bar{E}$ over *N* points, as given by:

(20) $$U_{o}=E_{min}{\left[\frac{1}{N}\sum_{i=1}^{N}\left(E_{i}\right)\right]}^{-1}=\frac{E_{min}}{\bar{E}}$$

where $E_{i}$ is the illuminance value at the $i_{th}$ point on the receiving plane. Here, the values of $U_{o}$ approaching 1 represent uniform illumination where, in general, the desired value is ≥ 0.7 [18]. Besides, in order to analyze the spread of illuminance, the interquartile range (IQR) of simulated data has also been considered. IQR is a statistical quantity that measures the dispersion of data by calculating the difference between its first and third quartiles, i.e., 3(N+1)/4−(N+1)/4. A high IQR value reflects a larger spread and vice versa.

On the other hand, the reduction in SNR fluctuations is evaluated using the standard deviation (σ). It is a statistical quantity that measures the dispersion of a data set relative to its average value (μ) and is given by:

(21) $$\sigma =\sqrt{\left(\frac{1}{N-1}\sum_{i=1}^{N}{\left|X_{i}-\mu \right|}^{2}\right)}$$

where $X_{i}$ is the quantity being evaluated (e.g., $P_{r}$ or SNR) and *N* is the number of points in the dataset. Ideally, the σ values should be close to zero.

### 3.5. Performance Evaluation Metrics

To measure the communication performance of an OBAN system, the following parameters have been used:

#### 3.5.1. RMS Delay Spread and Achievable Bit Rates

Apart from the LoS components, the power arriving at the PD also takes contributions from rays that are reflected off multiple paths. However, these multipath reflections lead to time spreading which limits the maximum achievable symbol rate without ISI. This time spreading is defined by the root mean square delay spread ($D_{rms}$), and it is given by [5]:

(22) $$D_{rms=}\sqrt[{2}]{\frac{\int {\left(t-\mu \right)}^{2}h^{2}\left(t\right)dt}{\int h^{2}\left(t\right)dt}}$$

where μ is the mean delay spread and h(t) is the total channel impulse response (CIR) that can be computed, as described in [5]. Using $D_{rms}$, the maximum supported data rate ($R_{b}$) for OOK-NRZ modulation format, without an equalizer, can be calculated as follows:

(23) $$R_{b}\leq \frac{1}{10D_{rms}}$$

#### 3.5.2. Bit Error Rate

In this study, since we have considered OOK-NRZ modulation such that light is transmitted to encode bit ‘1’ and no light transmission occurs for bit ‘0’, therefore, assuming the rectangular bit shape whose pulse duration equals the bit period, the BER is given by [10,11]:

(24) $$BER=Q(\sqrt{SNR})$$

where Q(.) is the Q-function which defines the error probability and it is given by [5]:

(25) $$Q\left(x\right)=\frac{1}{2\pi }\int_{x}^{\infty }e^{\frac{-u^{2}}{2}}du$$

For instance, the SNR that is required to achieve our targeted BER of $10^{-9}$ is 15.6 dB.

## 4. Result and Discussion

In order to analyze the impact of transmitter positions on the DL illuminance and SNR uniformities, ray-tracing simulations were carried out for twelve iterative arrangements (see Figure 3) in MATLAB^®^ environment using Barry’s benchmark ray tracing algorithm [35] and performance metrics related to illumination and communication were computed. Subsequently, the optimum case that was acquired with high uniformity of illuminance, low deviation in SNR, and lower average $D_{rms}$ was compared with coordinates obtained from Algorithm 1. These optimized locations were then compared with the conventional CLQ scheme. Even though the results for CLQ presented in [13] ignored the multipath dispersion effects which are critical from the communication perspective, to maintain a fair comparison in this study, we used the same simulation parameters (see Table 1) for both of the scenarios under consideration, except the LED locations. The outcomes of these simulations are discussed below, which have been sub-divided into two main sections to be analyzed from illumination and communication perspective. The illumination part is dedicated to DL only, whereas the communication part covers in-depth analysis of various parameters that affect the DL and UL communication performances in an OBAN system.

### 4.1. Illumination Performance

**Figure 5:**
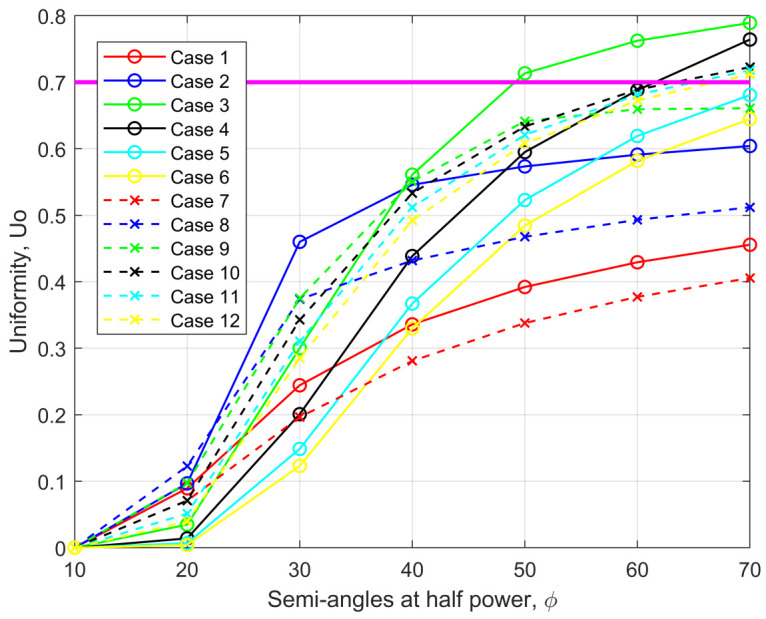
Uniformity of illuminance for cases I to XII at varying semi-angles at half power (SAAHP).

Figure 5 illustrates the illuminance uniformities obtained against varying semi-angles at half power (SAAHP) for all the twelve iterative cases. It can be seen that $U_{o}$ improves with increasing SAAHP and it is maximum at 70${}^{\circ }$. Since the LED without optics has SAAHP = 60${}^{\circ }$, we will limit analysis to 60${}^{\circ }$ in the following section. The CoIQ-based cases, which are closest to the desired uniformity value of 0.7, are the cases III, IV, X, XI, and XII. Here, it is important to state that, according to general indoor lighting placement rules, extreme corner lighting is not preferred and luminaires must be placed at least 0.6–0.9 m away from the corners to avoid wastage of light and unsightly shadows. Accordingly, even though cases XI and XII provide uniformity that is close to the threshold at 60${}^{\circ }$, these placement locations are not practical signifying that only cases III, IV, IX, and X can be considered (denoted hereafter as CoIQ-3, CoIQ-4, CoIQ-9, and CoIQ-10, respectively). Besides, when four transmitters are located close to the geometrical center of the room, uniformity is poor and it worsens with five transmitters, e.g., case-I and case-VII, which represent CLQ with four and five transmitters, respectively. Conversely, CoIQ-based arrangements with five transmitters provide better uniformity, which is relatively lesser with four transmitters. Table 2 summarizes the uniformity and IQR values that were obtained for these cases. The CoIQ cases outperform the CLQ based arrangement in terms of the uniformity of illuminance, with CoIQ-3 offering the best uniformity. CoIQ-10 has the least IQR dispersion, followed by CoIQ-3. However, to establish a comparison of our proposed corner-based placement scheme with the conventional technique, we will use CoIQ-9 as the worst-case scenario with the least uniformity of 0.6594.

**Figure 6:**
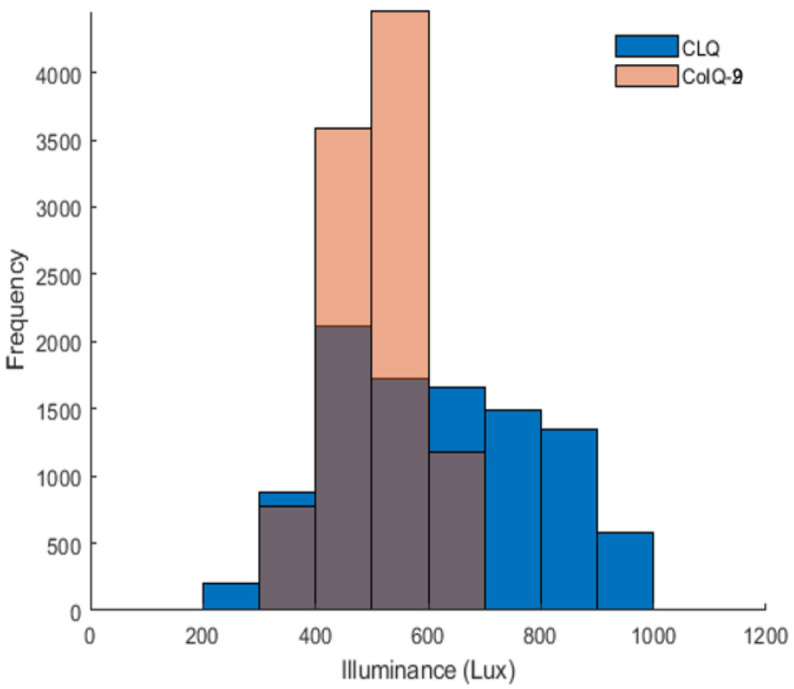
Illuminance histograms for CLQ and CoIQ-9.

Figure 6 depicts the illuminance histograms that were obtained for the two scenarios under consideration, i.e., CLQ and CoIQ-9. Both the cases satisfy the ISO based average illuminance requirements of 300-500 lux for indoor spaces with average values of 614.17 and 511.27 lux for CLQ and CoIQ-9, respectively. CLQ is spread over a wider range between 200–1000 lux which is reflected in its high IQR value of ∼ 295.82 lux (see Table 2). On the contrary, CoIQ-9 has a smaller spread, with values ranging between 300 and 600 lux and an IQR of only 111.23 lux. Besides, the uniformity values are 0.377 and 0.659 for CLQ and CoIQ-9, respectively. This indicates that even the worst-case scenario of the proposed arrangement has better uniformity than CLQ. Additionally, these values also validate that uniform distribution of illuminance is extremely poor in the conventional CLQ considering that the desired value is 0.7. It also implies that, relative to CLQ, the uniformity in CoIQ-9 has improved by 74.80%. Because CoIQ-9 had been assumed as the worst-case scenario, a further improvement in uniformity is also possible with other CoIQ-cases, i.e., 82.49% with CoIQ-10 and CoIQ-4 to a maximum of approximately 102% with CoIQ-3. However, the analysis of illuminance uniformity is not sufficient to remark on the communication quality of an optical link. Hence, the subsequent subsection will consider the effects of different lamp arrangements on the communication performance.

### 4.2. Communication Performance

A patient monitoring system requires the establishment of reliable bi-directional connections for information transfer; hence, we separately analyze the performance of our proposed technique for UL and DL scenarios.

#### 4.2.1. Downlink Analysis

##### *(a)* *Effects of Transmitter Locations on Received Power and SNR*

The characteristics of a VLC channel are affected by the change in Tx and Rx locations, the reflectivity of the surfaces, as well as the room size, such that the same mobile terminal may incur different performances at different locations inside the indoor space. Accordingly, realizing evenly distributed SNR is important for ensuring the same communication performance without having a dead zone. Table 3 presents the average RMS delay spread, mean, and standard deviation of $P_{r}$ and SNR for all the cases under consideration. The CoIQ-9 arrangement shows the least spread in $P_{r}$ and SNR, followed by CoIQ-10. However, CoIQ-4 experiences high deviation which is even higher than the conventional CLQ. Interestingly, CoIQ-3 is an exception here which, despite having the highest uniformity (see Table 2), exhibits high deviation. This shows that the number of luminaires and their placement plays a crucial role in determining the communication performance. Additionally, these results imply that high uniformity does not essentially guarantee least fluctuations in the SNR. Therefore, a complete lamp layout analysis must be performed prior to installation when VLC is desired.

**Figure 7:**
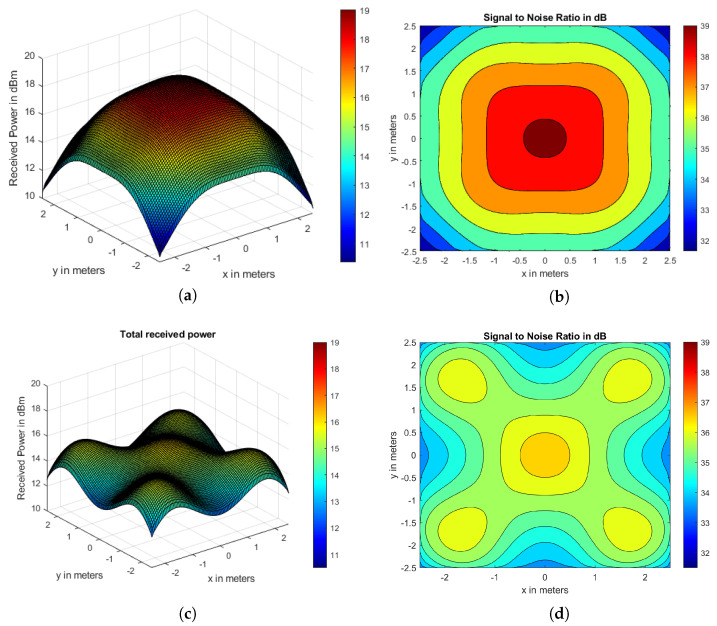
Received power for (**a**) CLQ (**c**) CoIQ-9 with respective SNR contours in (**b**,**d**).

The $P_{r}$ and SNR plots for CLQ and CoIQ-9 configurations are illustrated in Figure 7. The $P_{r}$ of CoIQ-9 is approximately restricted between 11 and 15.5 dBm with a variation of 4.5 dBm (Figure 7c) whereas the $P_{r}$ of CLQ is spread over a wider range of ∼8.5 dBm (Figure 7a). The higher spread in $P_{r}$ of CLQ reflects in a high σ of 1.706 dBm, whereas σ in CoIQ-9 has reduced to almost half the value of CLQ, as summarized in Table 3. Because SNR follows the same profile as $P_{r}$, the proportionality of reduced fluctuations in $P_{r}$ is also evident in SNR. For example, the SNR for CLQ is spread over ∼ 7.5 dB range (between 31.65 dB to 39.17 dB), whereas the SNR for CoIQ-9 varies over ∼3.3 dB range (between 33.42 to 36.74 dB) (see Table 3). This implies that the SNR range in CoIQ-9 reduces by almost 50%. The contour plot of CLQ shown in Figure 7b also shows that SNR is the highest at the center (∼39 dB) and tends to drop continuously by ∼1 dB for every 0.5 m away from the centre. The SNR plot for CoIQ-9 depicted in Figure 7d illustrates that the highest SNR value of ∼36.5 dB only covers a smaller area and the majority areas have SNR between 33.5–35.5 dB, implying that the SNR is more evenly spread in CoIQ-9 than in CLQ. This is also evident from Table 3, which indicates that σ of SNR reduces from 1.48 in CLQ to 0.678 in CoIQ-9, i.e. a reduction of ∼54.18% in the latter. Because the rearrangement of transmitters optimizes the uniformity of illuminance by 74% and reduces the fluctuations in SNR by 54% in CoIQ-9, it can be inferred that the locations of transmitters play a key role in determining the overall system performance. Moreover, the least SNR value is well above 15.6 dB, which is the minimum required to ensure a BER of $10^{-9}$.

##### *(b)* *Effects of Transmitter Locations on RMS Delay Spread*

**Figure 8:**
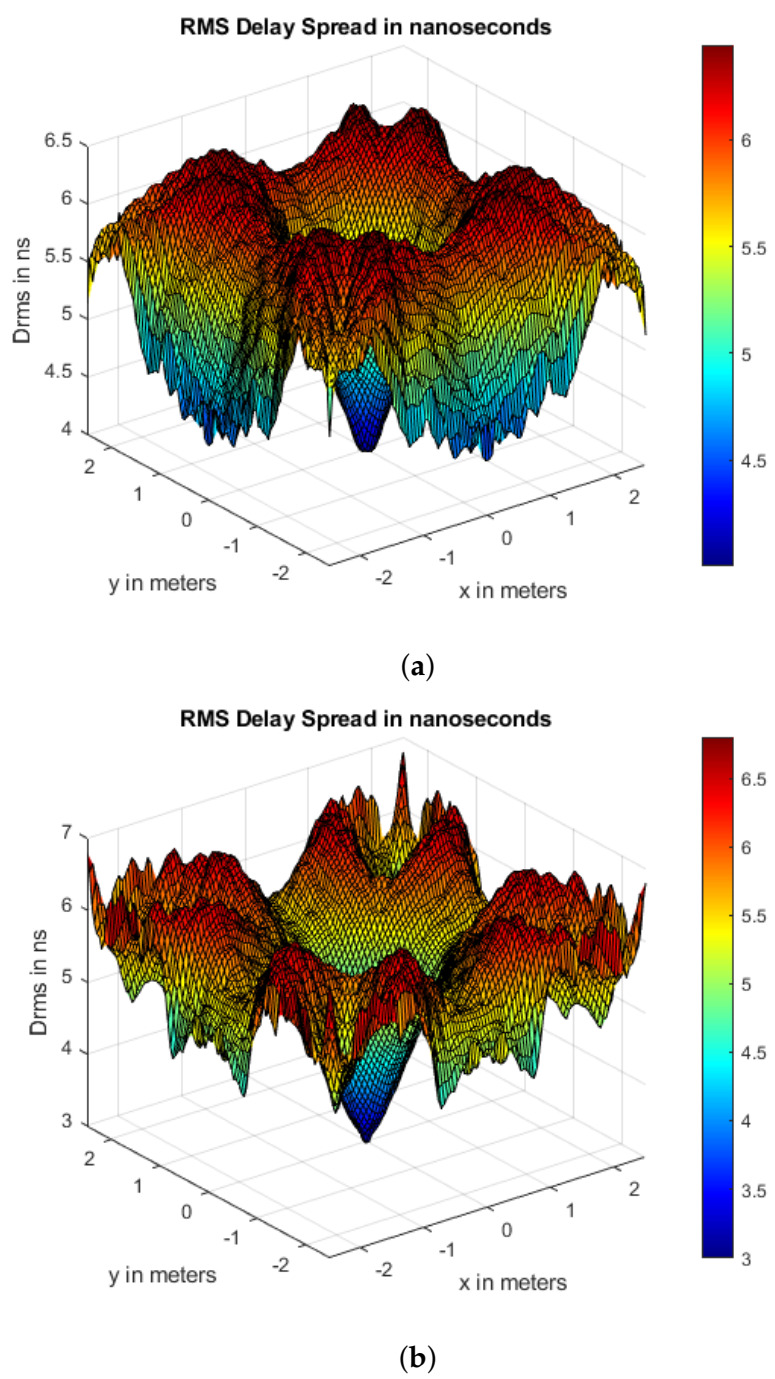
Root mean square (RMS) delay spread distribution for (**a**) CLQ and (**b**) CoIQ-9.

Figure 8 illustrates the $D_{rms}$ plots for CLQ and CoIQ-9 with values distributed symmetrically along the XY-receiving plane due to the square room geometry. For CLQ, the $D_{rms}$ values are lowest around the center of the room (Figure 8a). This is because all of these points are equidistant from the reflecting walls and the source, so that the difference in their traced path lengths is not very high. The maximum $D_{rms}$ value of ∼6.5 ns is present at the corner of the room, where this increase is attributed to large differences between LoS and reflecting paths from walls. For the CoIQ-9 arrangement (Figure 8b), $D_{rms}$ values are lowest at the center, moderate under each transmitter position and the edges, and maximum (∼7 ns) at other positions. Moreover, the average $D_{rms}$ is slightly lesser in CoIQ than in CLQ, with the values being 5.63 and 5.76 ns, respectively (see Table 3), due to which CoIQ has slightly better achievable data rates.

##### *(c)* *Effects of Varying Body Postures under Low-Lighting Conditions*

**Figure 9:**
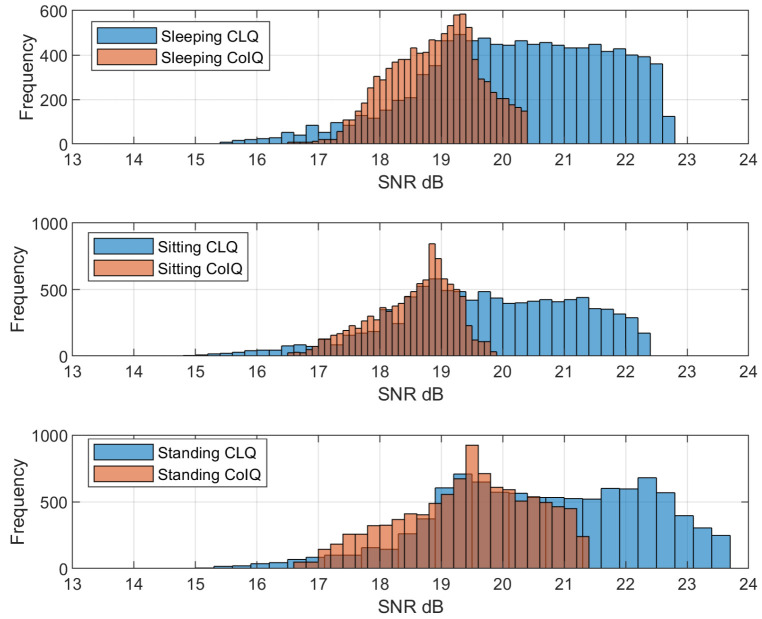
SNR histograms for sleeping, sitting and standing positions under low-intensity conditions in CLQ and CoIQ-9 arrangements.

All of the aforementioned results were based on the transmit power derived from a luminous intensity of 0.73 rcd, which produced high average illuminance levels of about 500 lux. However, in this section, we study the effect of low-lighting conditions on SNR distribution for standing, sitting, and sleeping positions when the average illuminance remains under 100 lux (required for sleeping scenarios). Figure 9 shows the histograms that were obtained for SNR at 10,000 different locations on the XY-receiving plane for all three postures. It can be observed that CoIQ has lesser spread in SNR than CLQ in all three scenarios. Moreover, the ${SNR}_{min}$ value for CoIQ for all the postures is approximately 16.5 dB which is sufficient to maintain a BER of $10^{-9}$, given that the threshold is 15.6 dB. On the contrary, CLQ offers ${SNR}_{min}$ of 15 dB, which does not ensure the minimum BER performance. This implies that, during low-lighting conditions, the user in CLQ may experience communication dips at several points in the room. Besides, we also determined the outage areas to further gauge the performance under low-lighting conditions, i.e., the regions where the SNR falls below the threshold level of 15.6 dB. The results have been summarized in Table 4 for various room dimensions. The outage area increases with the increasing room dimensions for both the configurations; however, the proposed-CoIQ technique will always have a lower outage area than CLQ. Based on these results and observations, it can be inferred that CoIQ will always achieve better illumination uniformity and lesser fluctuations in SNR when compared to the conventional CLQ scheme.

*Validation of the Proposed Heuristic Approach:* Since the analysis performed for illumination and communication (in Section 4.1 and Section 4.2, respectively) revealed that CoIQ-9 provides better performance than CLQ, we now validate that the proposed heuristic design formulae also yields optimum transmitter locations. Using (17)–(19), the optimized coordinates obtained from Algorithm 1 for 5 × 5 × 3 m${}^{3}$ dimensions are:

$$\begin{array}{ccccccc} {TP}_{0} & = & [ & \;\;\;0, & \;\;\;0, & \;1.5 & ]\\ {TP}_{1} & = & [ & -1.76, & -1.76, & \;1.5 & ]\\ {TP}_{2} & = & [ & -1.76, & \;\;1.76, & \;1.5 & ]\\ {TP}_{3} & = & [ & \;\;1.76, & \;\;1.76, & \;1.5 & ]\\ {TP}_{4} & = & [ & \;\;1.76, & -1.76, & \;1.5 & ] \end{array}$$

The resultant transmitter coordinates closely match with CoIQ-9 (see Figure 3), which implies that the proposed design formula provides optimum results. To corroborate the practicality of our method, Table 5 presents a comparison of CLQ and CoIQ for the results obtained against different room dimensions with a fixed number of transmitters. It can be observed that the performance metrics for illuminance and communication, especially the deviation in SNR, have improved substantially in CoIQ. Additionally, with exception of room sizes that are smaller in dimensions than 5×5×3 m${}^{3}$ (showing comparable average RMS delay spread), the $D_{rms}$ values in CoIQ are lesser than CLQ and will facilitate relatively better bit rates.

#### 4.2.2. Uplink Performance

**Figure 10:**
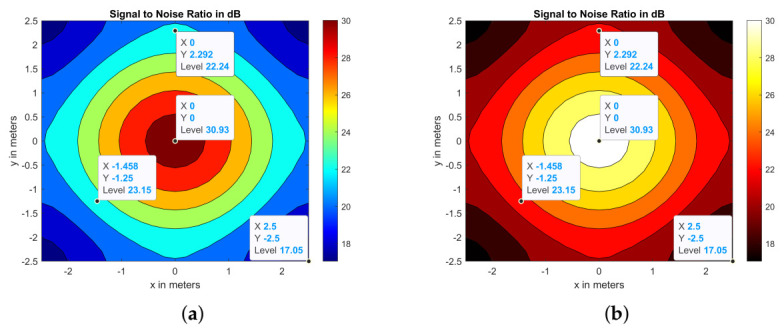
Reciprocity Principle: SNR distributions for DL and UL connections.

Having established in the previous section that the proposed-CoIQ placement technique outperforms CLQ from the illuminance as well as communication perspective, we now elaborate on how POR can be used to optimize the UL SNR uniformity. The principle assumes that, for a given set of parameters, the $P_{r}$ and SNR obtained when moving from point A → B in a linear channel will remain the same at the corresponding points if the transmitting and receiving devices are switched and direction of propagation is reversed, i.e., B → A. To illustrate this, standalone DL and UL SNR distributions were obtained via simulations for a simple one transmitter and one receiver scenario. For DL, the Tx was positioned on the ceiling at the center of the room with coordinates (0, 0, $L_{z}$/2), whereas the receiver was located along the XY-plane at a height of 0.85m. The SNR was calculated for 1089 different positions (see Figure 10a) and the deviation that was obtained for this arrangement was 3.44 dB (this high value can be attributed to the presence of only a single transmitter). On the other hand, Figure 10b shows the result for UL SNR when the transmitter and receiver positions were switched and the direction of propagation was reversed. The SNR deviation for UL was also found to be 3.44 dB. Moreover, the values at corresponding locations in the room are also similar, as illustrated in Figure 10a,b. Hence, if DL simulations were to be carried out individually for $TP_{1}\to RP$, $TP_{2}\to RP$, $TP_{3}\to RP$, $TP_{4}\to RP$, and $TP_{5}\to RP$, and super imposed, then the results obtained at corresponding points for $RP_{1}\leftarrow TP$, $RP_{2}\leftarrow TP$, $RP_{3}\leftarrow TP$, $RP_{4}\leftarrow TP$, and $RP_{5}\leftarrow TP$ would be the same. However, in a real scenario, the transmitters and receivers in UL may operate at different wavelengths (IR) and low transmit power values when compared to the DL (VLC) transceivers. Hence, UL analysis was performed with the transmit power and IR receiver responsivity values altered to 90 mW and 1A/W, respectively, in order to validate the practical application of reciprocity to our work. Additionally, the effect of ρ was considered, as the IR reflectivity is generally higher than that of VLC [36]. Table 6 summarizes the effect of each of these quantities on the UL SNR uniformities in terms of σ obtained for a typical hospital room size of 6.6 × 6.6 × 3 m${}^{3}$, as presented in [27]. It can be observed that $\sigma_{UL}$ is similar to the corresponding $\sigma_{DL}$ values for $P_{t}$ and γ (see Table 5), whereas the deviations are further reduced when the reflectivity is increased. Accordingly, it can be concluded that, even if the wavelength of operation is changed in an UL connection and the transmit power is low, the DL study can still be extended to determine the UL SNR uniformity using POR.

## 5. Conclusions

This paper highlighted the importance of the impact of LED lamp arrangements on DL SNR and illuminance uniformities. Accordingly, a heuristic design formula for obtaining optimal coordinates for generalized room dimensions and minimizing the fluctuations in SNR is outlined in the paper. It was shown that, by purposely relocating the transmitters around corners of the room according to the proposed placement technique, the SNR fluctuations can be reduced by ∼54% and the illuminance uniformity can be improved up to 102% when compared to the traditional approach. Moreover, the effects of low transmit power on DL communication were also presented. It was revealed that, for a worst-case scenario, where illuminance was lower than 100 lux, the SNR of the proposed scheme remains above the threshold level that is required to achieve a BER of $10^{-9}$, which is mandatory for achieving reliability in healthcare applications. Furthermore, the use of principle of reciprocity was demonstrated and validated using a single transmitter and single receiver scenario with the inference that DL uniformity analysis can be suitably used to estimate the UL SNR uniformity. Finally, the effects of UL parameters, like low transmit power, responsivity, and reflectivity, were analyzed to account for the change in wavelength of operation. It was concluded that SNR uniformity does not change with low power and responsivity, whereas it improves with increase in reflectivity. The future work includes the experimental verification of the proposed scheme in a realistic indoor environment. Moreover, the characterization of the channel impulse response for various receiver orientations in presence of human body, measurement of SNR in various environment, and demonstration of UL and DL OBAN communication will be carried out experimentally. Based on the measurements, further improvement in the simulation environment will be considered.

## Figures and Tables

**Table 1:** Summary of Simulation Parameters [5,11].

Parameters	Values
Room dimensions	5×5×3 m${}^{3}$
Reflection coefficients (all walls)	0.8
Area of differential element	0.0017 m${}^{2}$
**For Transmitter**	
Center luminous intensity per LED chip	0.73 Cd
Semi-angle at half-power	60${}^{\circ }$
Lambert’s order	1
LED luminous efficacy	90 lumens/watt
Number of LEDs per array	2500 (50×50)
Azimuth angle	0 ${}^{\circ }$
Elevation angle	−90 ${}^{\circ }$
**For Receiver**	
Field-of-View	90${}^{\circ }$
Physical Area	1 cm${}^{2}$
Gain of an optical filter	1
Refractive index of lens	1.5
Height (from the floor)	0.85 m
Bandwidth	20 MHz
Photodiode responsivity	0.54 A/W
Fixed Capacitance	112 pF/cm${}^{2}$
Open loop gain	10
Temperature	300 K
Noise Bandwidth factor (I${}_{2}$)	0.562
Noise Bandwidth factor (I${}_{3}$)	0.0868
FET transconductance	30 mS
FET noise factor	1.5
Dark current (I${}_{d}$)	2 pA
N${}_{x}$	100
N${}_{y}$	100
N${}_{z}$	90

**Table 2:** Performance Metrics for Illumination.

Lamp Placement Scenario	Illuminance	
	Uniformity	IQR (Lux)
CLQ	0.377	295.82
CoIQ-10	0.688	70.59
CoIQ-9	0.659	111.23
CoIQ-4	0.688	109.43
CoIQ-3	0.762	75.37

**Table 3:** Performance Metrics for Communication.

Lamp Placement Scenario	Communication						
	RMS Delay Spread		Received Power (dBm)		SNR (dB)		
	Average $D_{\mathrm{rms}}$ (ns)	Average Achievable Bitrates (Mbps)	Average	Standard Deviation	Min	Max	Standard Deviation
CLQ	5.76	17.36	16.00	1.71	31.65	39.17	1.48
CoIQ-10	5.44	18.38	13.68	0.92	32.43	35.93	0.80
CoIQ-9	5.63	17.79	14.70	0.78	33.42	36.74	0.68
CoIQ-4	5.00	20.00	10.12	1.99	28.57	34.71	1.74
CoIQ-3	5.56	17.98	11.62	1.38	30.52	34.94	1.20

**Table 4:** Percentage Outage Areas for CLQ and CoIQ.

	Outage Area (%)	
**Room size** **(m${}^{3}$)**	**CLQ**	**CoIQ**
5 × 5 × 3	0.08	0
5.5 × 5.5 × 3	2.72	0.56
6 × 6 × 3	10.68	4.52

**Table 5:** Downlink performance of CoIQ and CLQ for different room dimensions.

Room Size (m${}^{3}$)	CLQ				Proposed-CoIQ			
	Uniformity	Deviation in SNR (dB)	Average $D_{\mathrm{rms}}$ (ns)	Average Achievable Data Rates (Mbps)	Uniformity	Deviation in SNR (dB)	Average $D_{\mathrm{rms}}$ (ns)	Average Achievable Data Rates (Mbps)
2.5 × 2.5 × 3	0.7111	0.8476	3.30	30.30	0.8612	0.6792	3.57	28.011
4 × 4 × 3	0.5209	1.2240	5.38	18.58	0.8273	0.5962	5.34	18.72
6.6 × 6.6 × 3	0.3329	1.8730	8.64	11.57	0.5370	1.1154	7.04	14.20
7 × 7 × 3	0.3115	2.0011	8.87	11.27	0.5410	1.2346	7.30	13.69
8 × 8 × 3	0.2641	2.3652	9.14	10.94	0.4596	1.9624	7.85	12.73

**Table 6:** Uplink performance of CLQ and CoIQ for given room dimensions

	CLQ			Proposed CoIQ		
	Deviation in SNR (dB)					
Room Size (m${}^{3}$)	$P_{t}$ = 90 mW	ρ = 0.9	γ = 1.01 A/W	$P_{t}$ = 90 mW	ρ = 0.9	γ = 1.01 A/W
6.6 × 6.6 × 3	1.87	1.75	1.87	1.11	1.06	1.11

## Data Availability

Not applicable.
